# Induction of axial chirality in divanillin by interaction with bovine serum albumin

**DOI:** 10.1371/journal.pone.0178597

**Published:** 2017-06-02

**Authors:** Diego Venturini, Aguinaldo Robinson de Souza, Ignez Caracelli, Nelson Henrique Morgon, Luiz Carlos da Silva-Filho, Valdecir Farias Ximenes

**Affiliations:** 1 Department of Chemistry, Faculty of Sciences, UNESP—São Paulo State University, Bauru, São Paulo, Brazil; 2 BioMat, Department of Physics, Federal University of São Carlos, São Carlos, São Paulo, Brazil; 3 Department of Physical Chemistry, Institute of Chemistry, Campinas State University (UNICAMP), Campinas, São Paulo, Brazil; Islamic Azad University Mashhad Branch, ISLAMIC REPUBLIC OF IRAN

## Abstract

Vanillin is a plant secondary metabolite and has numerous beneficial health applications. Divanillin is the homodimer of vanillin and used as a taste enhancer compound and also a promissory anticancer drug. Here, divanillin was synthesized and studied in the context of its interaction with bovine serum albumin (BSA). We found that divanillin acquires axial chirality when complexed with BSA. This chiroptical property was demonstrated by a strong induced circular dichroism (ICD) signal. In agreement with this finding, the association constant between BSA and divanillin (3.3 x 10^5^ mol^-1^L) was higher compared to its precursor vanillin (7.3 x 10^4^ mol^-1^L). The ICD signal was used for evaluation of the association constant, demonstration of the reversibility of the interaction and determination of the binding site, revealing that divanillin has preference for Sudlow’s site I in BSA. This property was confirmed by displacement of the fluorescent markers warfarin (site I) and dansyl-*L*-proline (site II). Molecular docking simulation confirmed the higher affinity of divanillin to site I. The highest scored conformation obtained by docking (dihedral angle 242°) was used for calculation of the circular dichroism spectrum of divanillin using Time-Dependent Density Functional Theory (TDDFT). The theoretical spectrum showed good similarity with the experimental ICD. In summary, we have demonstrated that by interacting with the chiral cavities in BSA, divanillin became a atropos biphenyl, i.e., the free rotation around the single bound that links the aromatic rings was impeded. This phenomenon can be explained considering the interactions of divanillin with amino acid residues in the binding site of the protein. This chiroptical property can be very useful for studying the effects of divanillin in biological systems. Considering the potential pharmacological application of divanillin, these findings will be helpful for researchers interested in the pharmacological properties of this compound.

## Introduction

Vanillin, a component of vanilla, is one of the most widely used flavors in the world and has numerous applications in food, beverage and pharmaceutical industries [1]. Vanillin is a plant secondary metabolite found in several essential plant oils as Vanilla planifolia, Vanilla tahitensis, and Vanilla pompona [2]. Vanillin has numerous beneficial health applications due to its antioxidant [3], neuroprotective [4] and anticancer properties [5].

Differently of vanillin, its homodimer, divanillin was much less investigated. However, the pharmacological potential of this compound is quite promisor, as can be verified in a very recent publication that showed its capacity to decrease the metastatic potential of human cancer cells by inhibiting the FAK/PI3K/Akt signaling pathway [6]. Other application of divanillin is its use as a taste enhancer which imparts pleasant impressions of creaminess to food [7].

From the point of view of its molecular structure, vanillin has great similarity with apocynin, and its dimer, diapocynin [8], is similar to divanillin. This was one of the reasons behind our interest in the later molecule. Indeed, there is a great chance that divanillin can be as active as diapocynin regarding its biological activities. It's worth remembering that apocynin, an inhibitor of the NADPH oxidase enzymatic complex, has numerous pharmacological effects [9,10], and there is increasing evidence that diapocynin is also a promissory drug [11–13].

Divanillin is an ortho-substituted biphenyl system, and, as such, it is reasonable to suspect the existence of axial chirality for this compound, i.e., the existence of energetic barrier that could impede the rotation around the central single bond that connect the aromatic rings. The consequence would be the existence of stable axial stereoisomers. This phenomenon is known as atropisomerism or axial chirality and divides the biaryl system in two categories: atropos systems, from a (not) tropos (to turn), when the rotation is restricted due to steric hindrance; and tropos system, when racemization is observed due to the low energy barrier. In other words, only atropos biphenyls may exist as a pair of stable enantiomers.

Human serum albumin (HSA) is the most abundant protein in the human bloodstream and has many physiological functions, including a significant contribution for the colloidal osmotic pressure and as a carrier and depot protein for metabolites and xenobiotics [14]. It is a heart-shaped protein composed of three structurally similar domains I-III and two subdomains named A and B. To transport pharmaceutical drugs, HSA has two main binding sites located in hydrophobic cavities in subdomain IIA and IIIA. These binding sites are known as site I, which is a pocket in subdomain IIA and site II in subdomain IIIA [15]. The binding of a ligand in a protein may alters the spectroscopic properties of the ligand, of the protein, or both. This is, for instance, the foundation of the widely applied procedure for measurement of association constants based on intrinsic fluorescence quenching of albumin provoked by the complexation with the ligand [16]. Others well established methods for studding protein-ligand interactions include isothermal titration calorimetry, light scattering, chromatographic and circular dichroism techniques [17–25]. In the last case, an optically inactive compound may became optically active when fixed in the binding site of a protein. This phenomenon is very useful as an analytical tool for studying the interaction between ligands and proteins. It is known as induced chirality and is experimentally detected by the appearance of a new circular dichroism signal, which originally was not present in the protein or in the free ligand [17]. How proposed by Gawronski and Grajewski there are two different conditions for induction of chirality: induction of a dominating chiral structure of the achiral molecule or induction of a chiral arrangement of the electric dipole transition moments between the relevant chromophores of the achiral (guest) and chiral (host) molecules [26]. Regardless its origin, the induction of chirality by host-guest interaction leads to a new circular dichroism signal, which is known as induced circular dichroism (ICD) and can be useful for studying the interaction between proteins and ligands. In particular, for the characterization of the binding sites of new bioactive compounds in albumin, the application of ICD is useful and two approaches can be used: i) the studied compound is susceptible to the induction of chirality. In this case, the use well-established competitor ligands for site I or II can be applied for searching the alteration in the ICD signal and, consequently, indicates the site of higher affinity for the studied compound. ii) the studied compound is not susceptible to induction of chirality. In this case, the use of drugs that are well-stablished ligands of site I or II and susceptible to ICD are used to evaluate the effect of the competitor studied compound. For instance, phenylbutazone, diazepam and bilirubin as site markers for site I, II and III, respectively [17, 26, 27] and dansylglycine for site II [28] are all susceptible to ICD. For these reasons, here we aimed to study the features of divanillin as a ligand of bovine serum albumin (BSA). We hypothesized that due to its biphenyl moiety, divanillin could be susceptible to induction of chirality by interacting with BSA. This study provides the first experimental finding about the interaction of this potential bioactive compound with albumin.

## Material and methods

### Chemicals and reagents

Bovine serum albumin fatty acid free and essentially globulin free (A7030), ibuprofen, warfarin, phenylbutazone, naproxen, vanillin, potassium persulfate and ammonium iron (II) sulphate hexahydrate were purchased from Sigma-Aldrich Chemical Co. (St. Louis, MO, USA). Stock solutions of the pharmaceutical compounds and divanillin (10 mmol L^-1^) were prepared in dimethyl sulfoxide. Working solutions were prepared by dilution the stock solution in 50 mmol L^-1^ phosphate buffer at pH 7.0. BSA was dissolved in 50 mmol L^-1^ phosphate buffer at pH 7.0 to give a 1 mmol L^-1^ stock solution and its concentration measured by its absorbance (ε _280nm_ = 43,291 mol^-1^ L cm^–1^) [29].

### Divanillin: Synthesis and characterisation

Divanillin was prepared as previously described for the preparation of diapocynin with modifications [30]. Vanillin (0.912 g, 6 mmol) was dissolved in 200 mL of hot water. Then, the heating was turned off and ammonium iron(II) sulfate hexahydrate (118 mg, 0.3 mmol) and potassium persulfate (811 mg, 3.0 mmol) were added and stirred for 30 min. The precipitated product was filtered and washed with cold water. The product was re-dissolved by adding sodium hydroxide (50 mL, 4 mol L^-1^) and filtered. The solution was acidified by adding hydrochloric acid (50 mL, 4 mol L^-1^). The precipitate was filtered and washed with cold water. The product was dried in vacuum over phosphorus pentoxide, yielding 0.64 g (71%) of a yellow pale solid. The purity was confirmed by HPLC analysis in line with a diode array detector set at 254 nm (Jasco, Tokyo, Japan). The analyses were carried on a Luna C18 reversed-phase column (250 x 4.6 mm, 5 μm) using solvent A (aqueous formic acid 0.1%) as a mobile phase and solvent B (formic acid 0.1% in acetonitrile). The gradient was solvent A 90% to 10% in 22 min. The flow rate was 1 mL min^-1^. NMR spectra were obtained using DMSO- D_6_ as solvent and internal reference for ^1^H and ^13^C (Bruker DRX 400 spectrometer, MA, USA): ^1^H NMR (400 MHz) δ (ppm): 9.80–9.88 (m, 2OH), 9.82 (s, 2CHO), 7.43–7.45 (m, 4 CH-Ar), 3.94 (s, 2 OCH_3_). ^13^C NMR (100 MHz) δ(ppm): 191.2 (2CHO), 150.4 (2C), 148.1 (2C), 128.1 (2CH), 127.8 (2C), 124.6 (2C), 109.2 (2CH), 56.0 (2OCH_3_) (S1 and S2 Figs). These results are consistent with those previously reported [31].

### Fluorescence quenching experiments

The complexation of divanillin with BSA was evaluated by the protein intrinsic fluorescence quenching. The fluorescence experiments were performed with the following settings: excitation at 295 nm and emission in the range 310 nm to 450 nm. The slit widths were 5 nm for both excitation and emission wavelengths. The experiments were performed using a 3 mL quartz cuvette with a 10 mm path length and magnetically stirred during the measurements. Fluorescence quenching experiments were performed by titration of BSA (5 μmol L^-1^) with divanillin (0–6 μmol L^-1^) in 50 mmol L^-1^ phosphate buffer, pH 7.0, at different temperatures. After each addition, the protein/ligand mixtures were incubated for 2 min before the fluorescence measurements. The fluorescent intensities were corrected for the inner filter effect caused by attenuation of the excitation and emission signals provoked by the absorption of divanillin using Eq 1 [32]. In this equation *F*_*corr*_ and *F*_*obs*_ are the corrected and observed fluorescence intensities, respectively. *Ab*_*ex*_ and *Ab*_*em*_ are the absorptions of the mixture at excitation (295 nm) and emission wavelengths (343 nm), respectively. The absorbance and fluorescence spectra were measured using a Perkin Elmer Lambda 35 UV−visible spectrophotometer and Perkin Elmer LS 55 spectrofluorimeter, respectively (Shelton, CT, USA). The linear and non-linear fittings for Stern-Volmer and association constants were obtained using the GraphPad Prism version 5.00 for Windows (GraphPad Software, San Diego California USA).

$$F_{corr}=F_{obs}\times 10^{\left(Ab\;ex+Ab\;em\right)/2}$$ (1)

### Characterisation of binding sites

The characterisation of the binding site was evaluated by displacement of the fluorescent site markers warfarin [33] and dansylproline [34]. For the studies with warfarin (site I), the spectrofluorimeter was adjusted to excitation at 310 nm and emission in the range 330–450 nm. The experiments were performed by the addition of varying amounts of the divanillin or the pharmaceutical compounds to a mixture of 5 μmol L^-1^ BSA and 5 μmol L^-1^ warfarin in 50 mmol L^-1^ phosphate buffer at pH 7.0. For the studies with dansylproline (site II) the excitation was set at 360 nm and emission in the range 400–600 nm. The experiments were performed by the addition of varying amounts of the divanillin or the pharmaceutical compounds to a mixture of 10 μmol L^-1^ BSA and 20 μmol L^-1^ dansylproline in 50 mmol L^-1^ phosphate buffer at pH 7.0. The mixtures were incubated for 2 min at 25°C before the measurements.

### Circular dichroism experiments: ICD studies

The protein-ligand interaction was studied by the induction of chirality in divanillin provoked by its binding in BSA. The CD studies were performed in a Jasco J-815 spectropolarimeter (Jasco, Japan) equipped with a thermostatically controlled cell holder. The spectra were obtained with 1 nm step resolution, response time of 1 s and scanning speed of 50 nm/min. A 3 mL quartz cuvette with a 10 mm path length and a magnetic stirrer were used for the measurements in the near-UV-CD range. The baseline (50 mmol L^-1^ phosphate buffer) was subtracted from all measurements. The experiments were performed by titration of BSA (30 μmol L^-1^) with divanillin (0–90 μmol L^-1^) in 50 mmol L^-1^ phosphate buffer, pH 7.0, at different temperatures. The ICD was used to monitor the displacement of divanillin from the its binding sites in BSA using well-accepted pharmaceutical drugs that are specific ligands for site I (phenylbutazone and warfarin) and for site II (naproxen and ibuprofen). The experimental condition was equimolar amounts of BSA:divanillin (30 μmol L^-1^) and increasing amounts of the drugs (0–90 μmol L^-1^) [35].

### Theoretical studies

In this study, we employed the quantum chemical formalism at the Time-Dependent Density Functional Theory (TDDFT) theory to understanding the experimental results. The search for the most stable conformation adopted by divanillin, in the gas phase, was carried out employing M06-2X/6-31G(d) hybrid exchange-correlation functional. All computer simulations were done in the GridUnesp supercomputer facilities, which is composed of 256 SUN X4150 servers with 2048 cores (Intel Xeon 2.83 GHZ), with 4096 GB of RAM memory (2 GB per core) and an infiniband 4X DDR (20 Gbps) connection. The storage capacity of these system is 36 TB through DAS optical fibre (StorageTek 6140) and 96 TB at four SUN X4500 servers. The Gaussian09 suite of programs was employed to obtain the geometric and energy parameters for divanillin [36]. The computer simulation of the ECD spectrum was carried out using the CAM-B3LYP, a hybrid exchange–correlation functional that combines the hybrid qualities of B3LYP and the long-range correction, and 6–311++G(2d,p) basis set. The solvent was modelled using the Polarizable Continuum Medium (PCM) using the dielectric constant of ethanol (ε = 24.852).

### Docking simulations

The crystallographic structure used was that of BSA in complex with naproxen available at the Protein Data Bank (PDB) access code 4OR0 [37]. Simulations were carried out using GOLD 5.2 (Genetic Optimization for Ligand Docking), a software based on a genetic algorithm to explore the ligand conformational space and fitness function GoldScore [38,39]. Studies in each site binding were carried out independently for divanillin. Before performing the docking studies, the crystallographic ligands were removed from their site and the calculations performed in a sphere of 10 Å radius centered in the cavity where the crystallographic ligand was located.

### Statistics

Results were shown as the means of three independent experiments.

## Results and discussion

### Synthesis, characterisation, determination of binding and thermodynamic constants

**Fig 1:**
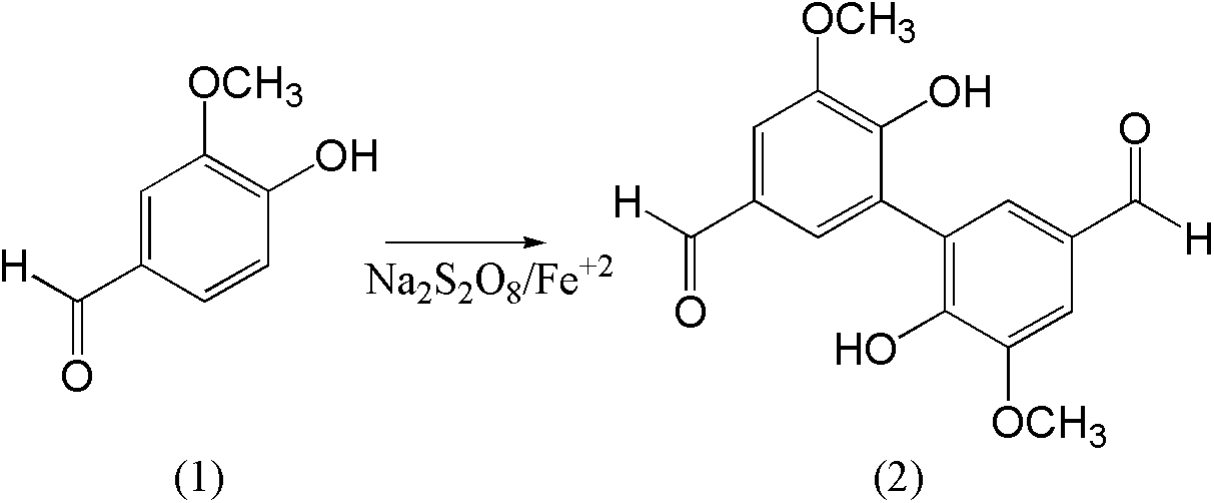
Molecular structure of vanillin (1) and divanillin (2).

Divanillin (6,6′-dihydroxy-5,5′-dimethoxy-[1,1′-biphenyl]-3,3′-dicarboxaldehyde) was synthesised by ferrous sulfate-catalysed oxidation of vanillin (4-hydroxy-3-methoxybenzaldehyde) using sodium persulfate as the oxidising agent (Fig 1). Its purity (> 95%) was evaluated by HPLC and its identity by NMR. The HPLC analysis showed a higher retention time to divanillin (14.0 min) compared to vanillin (9.1 min), which is a consequence of its higher hydrophobicity (S1 and S2 Figs). The binding affinity of divanillin to BSA was initially evaluated by the protein intrinsic fluorescence quenching caused by its interaction with this potential ligand. This photophysical phenomenon can be explained by collisional deactivation (dynamic quenching) and/or formation of a ground-state complex (static quenching) between the protein and ligand [40].

**Fig 2:**
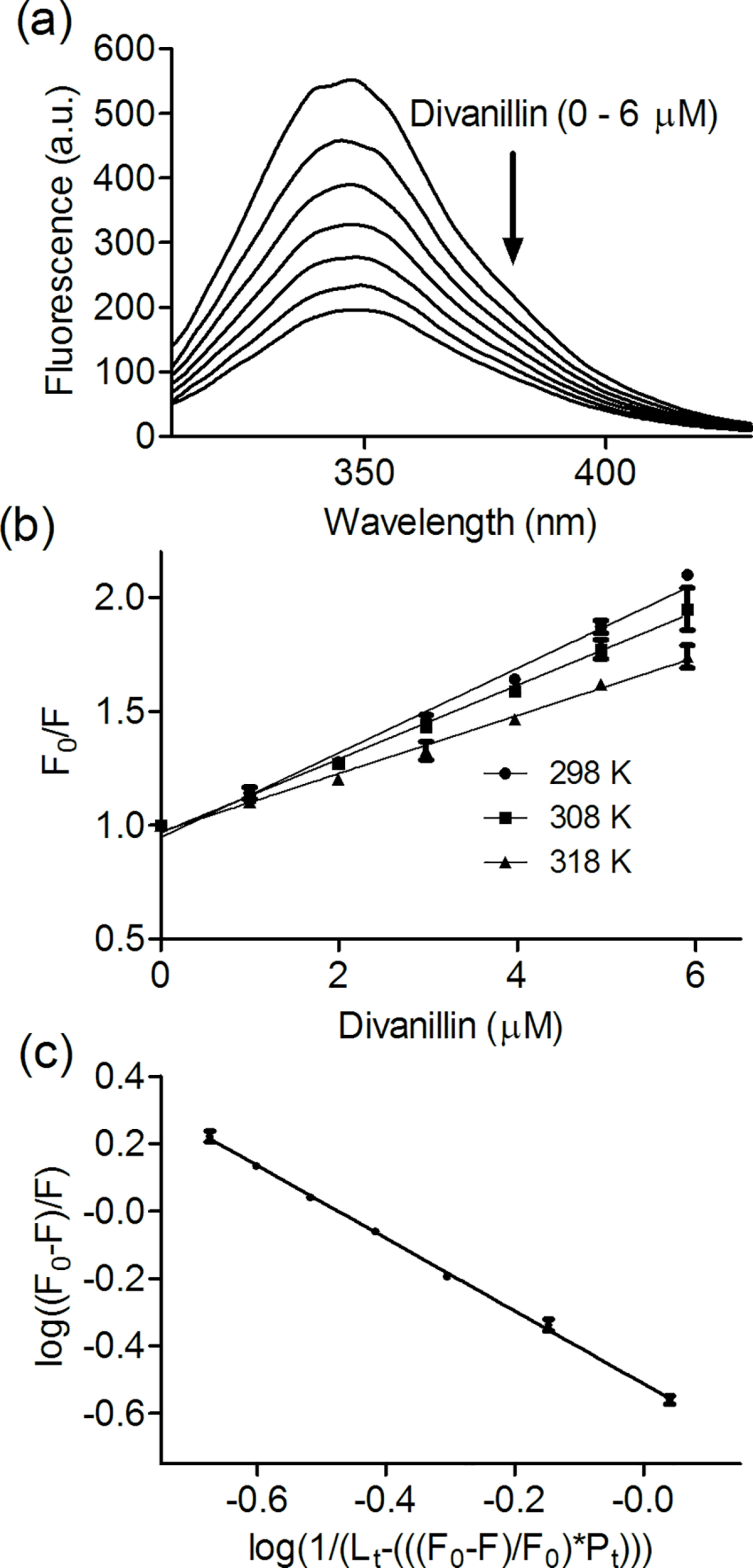
Fluorescence quenching of BSA by divanillin and determination of Stern-Volmer and association constants. **(a)** Emission spectra of 5 μmol L^-1^ BSA in the absence or presence of divanillin (0–6 μmol L^-1^) in 0.05 mol L^-1^ phosphate buffer pH 7.0 at 298 K. **(b)** Stern-Volmer plots at different temperatures (λ_ex_ = 295 nm, λ_em_ = 343 nm). **(c)** Double logarithmic fitting to obtain the association constant. The results are the average and SD of three experiments.

Fig 2A shows that the fluorescence intensity of BSA was strongly decreased by the addition of divanillin. Therefore, aiming to evaluate the mechanism of interaction, the experimental data were mathematically treated using the Stern-Volmer equation. According to this mathematical model, the linear correlation obtained using the Stern-Volmer equation (Eq 2, Fig 2B) is an indication that only one mechanism of quenching is involved in suppression of the fluorescence in the experimental condition used here, being dynamic or static [40]. In this equation, *F*_*0*_ and *F* are the fluorescence intensity of BSA in the absence and presence of the divanillin, respectively; *K*_*sv*_ is the Stern-Volmer constant; *kq* is the bimolecular quenching constant; τ^0^ is the average lifetime of the fluorophore tryptophan in (BSA) in the absence of the divanillin, and *[Q]* is the concentration of divanillin. Before fitting the data to Stern-Volmer equation, the fluorescent intensities were corrected for the inner filter effect as presented in the material and methods section. It is worth of note that the inner filter effect is provoked by attenuation of the excitation and emission signals due to the absorption of the study compounds [32].

$$\frac{F_{0}}{F}=1+K_{SV}\times \left[Q\right]=1+k_{q}\times \tau^{0}\times \left[Q\right]$$ (2)

The magnitude of the obtained Stern-Volmer constant (1.73 x 10^5^ mol^-1^ L at 298 K) revealed the strong association between divanillin and BSA (Fig 2 and Table 1). From this value and assuming τ^0^ for tryptophan in albumin as ~ 6 x 10^−9^ s [41], the *k*_*q*_ resulted in approximately 2 × 10^13^ mol^−1^ L s^−1^. As this value is higher than the maximum scatter collision quenching constant (2 × 10^10^ mol^−1^ L s^−1^), the quenching mechanism can be considered a static process [40]; in other words, the quenching must be caused by the formation of a ground-state complex between divanillin and BSA. Furthermore, additional evidence that the fluorescence quenching resulted from the complexation between divanillin and BSA was obtained by measuring the effect of temperature on the Stern-Volmer constant. The results depicted in Fig 2B and Table 1 show that the interaction was weakened at higher temperatures, which is a typical characteristic of static quenching provoked by the formation of the ground-state complex [40].

**Table 1:** Stern-volmer and bimolecular quenching constants for the interaction between divanillin and BSA.

T (K)	*K*_*sv*_ (10^5^ M^-1^)	R^2^	*k*_*q*_ (10^13^ s^-1^)
**298**	1.73 ± 0.06	0.9869	1.73 ± 0.06
**308**	1.54 ± 0.04	0.9887	1.54 ± 0.04
**318**	1.21 ± 0.04	0.9882	1.21 ± 0.04

The bimolecular quenching constant indicated that divanillin is a ligand of BSA. Therefore, the quenching experimental data can be used for determination of the association constant (*K*_*a*_) using a double logarithmic equation (Fig 2C and Eq 3). This mathematical treatment was chosen because the concentration of free ligand in equilibrium cannot be assumed as equal to the total ligand added during the titration, which is the case here, since only approximately equimolar amount of divanillin and BSA were used in our experimental setup [42]. In this equation: *F*_*0*_: fluorescence in the absence of divanillin; *F*: fluorescence in the presence of divanillin; *P*_*t*_: total BSA concentration; *L*_*t*_: total added divanillin; *K*_*a*_: association constant; *n*: stoichiometry of the binding.

$$log\frac{F_{0}-F}{F}=n\times logKa-n\times log\frac{1}{\left[L_{t}\right]-\frac{F_{0}-F}{F_{0}}\left[P_{t}\right]}$$ (3)

Fig 2C shows the excellent linear correlation and Table 2 the values of *K*_*a*_ obtained for the complexation between BSA and divanillin at different temperatures. These values reinforce the strong association of divanillin with BSA (3.3 x 10^5^ mol^-1^ L), which is comparable to those obtained for pharmaceutical compounds that are well-stablished as ligand of albumin, for instance: warfarin (4.2 x 10^5^ mol^-1^ L), naproxen (3.8 x 10^5^ mol^-1^ L), phenylbutazone (1.3 x 10^5^ mol^-1^ L) and mesalazine (2.5 x 10^5^ mol^-1^ L) [43–46]. Moreover, it is about one order of magnitude higher compared to the value reported to vanillin (7.7 x 10^4^ mol^-1^ L) [47]. It is worth of note that this value was obtained for the binding of vanillin to HSA; hence, for a more effective comparison with our results, we also measured the binding of vanillin with BSA (S3 Fig). At 298 K the association constant of vanillin with BSA was 7.3 x 10^4^ mol^-1^ L, which is in agreement with the reported value to HSA [47], and reinforce the higher affinity of BSA to divanillin compared to vanillin. Finally, Table 2 also shows that *n* value for the complexation of divanillin was close to one, which is indicative of a 1:1 stoichiometry. However, this result does not discard the possibility of more than one binding site and/or sites with less affinity could be also occupied using a large excess of divanillin.

**Table 2:** Association constant and thermodynamic parameter for the interaction between divanillin and BSA.

T (K)	*K*_*a*_ (10^5^ mol^-1^ L)	*n*	R^2^	ΔH° (kJ mol^-1^)	ΔS° (J mol^-1^)	ΔG° (kJ mol^-1^)
**298**	3.33	0.9953	0.9901	-19.42	+40.8	-31.5
**308**	2.83	1.018	0.9903			-32.1
**318**	2.03	1.054	0.9916			-32.3

Aiming a better comprehension of the non-covalent forces involved in the complexation of divanillin, the thermodynamic parameters for binding were obtained by plotting the natural logarithm of the association constants (ln *K*_*a*_) against the absolute reciprocal temperature (1/T) using the Van`t Hoff equation (Eq 4) (S4 Fig). From the values of enthalpy (ΔH°) and entropy changes (ΔS°); the free energy change (ΔG°) for the binding were calculated using the free energy equation (Eq 5). Where R is the gas constant (8.31 J mol^− 1^ K^− 1^) and T is the absolute temperature.

$$\ln K_{a}=-\frac{\Delta H{}^{\circ}}{R}\times \frac{1}{T}+\frac{\Delta S{}^{\circ}}{R}$$ (4)

ΔG°=ΔH°−TΔS° (5)

Table 2 displays the obtained values of ΔH° and ΔS° and the ΔG° calculated at three different temperatures. From these data, the efficacy of BSA as a potential carrier of divanillin was proved by the negative ΔG°, which shows the spontaneity of the binding. The affinity can be explained by the relatively large and negative ΔH° (-19.4 kJ mol^-1^), which have been demonstrated to be correlated with the formation of hydrogen bonds and Van der Waals forces and by the positive ΔS° (40.8 J mol^-1^ K^-1^), an indicative of the importance of hydrophobic interactions for the formation of the complex [48,49]. A comparison can be also made with the values reposted for vanillin ΔH° (-20 kJ mol^-1^) and ΔS° (5.8 J mol^-1^ K^-1^) [47]. These results suggest that hydrophobic interactions can be the major force that differentiate and may explain the higher efficacy of divanillin as a ligand of albumin compared to its precursor vanillin. Corroborant with that, the hydrophobicity index of divanillin (log P = 2.17) is almost twice compared to vanillin (log P = 1.26). Therefore, it is reasonable to propose that the layers of water molecules around divanillin must be more organized compared to vanillin in the solvent bulk, and consequently, the displacement of the dimeric molecule to the protein cavity must be associate with a higher positive entropy change. It is worth of note that the molecular hydrophobicities of vanillin and divanillin were calculated based on their log P values (partitioning coefficient in n-octanol/water) based on Crippen’s fragmentation [50].

### Induction of chirality in divanillin by binding with BSA

**Fig 3:**
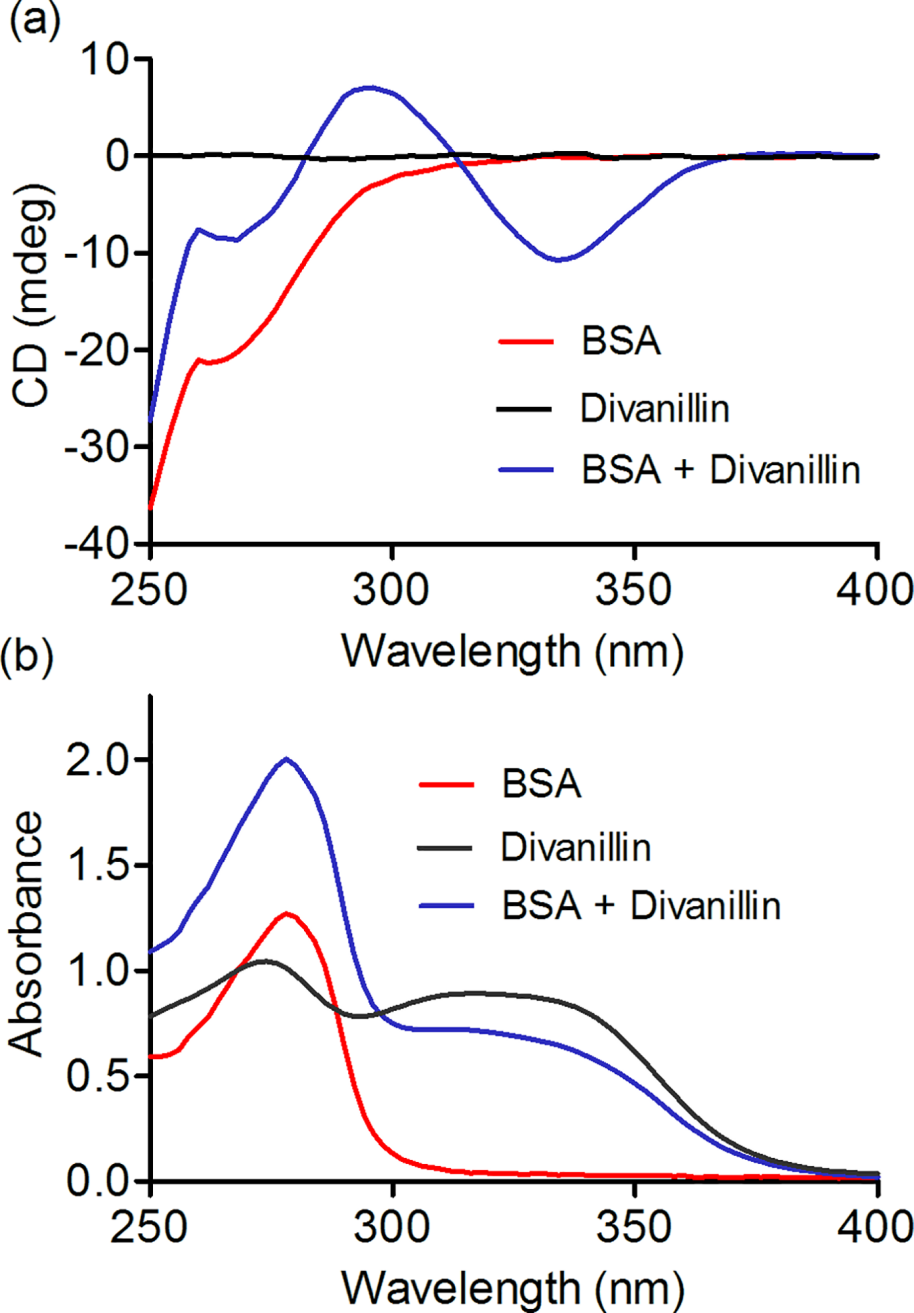
Induction of circular dichroism signal in divanillin provoked by interaction with BSA. **(a)** ICD and **(b)** absorbance spectra of BSA in the presence or absence of divanillin. The mixtures consisted of 30 μM BSA and 30 μM divanillin in 0.05 M phosphate buffer at pH 7.0.

Divanillin is an ortho-substituted biphenyl system; hence, it is reasonable to suspect the existence of axial chirality. To evaluate this putative chiroptical property, its CD spectrum was measured as indicated in Fig 3. How can be observed, in the UV-visible region of absorption, no signs of chirality were obtained to free divanillin dissolved in buffered aqueous solution. However, the addition of BSA provoked the appearance of a clear CD spectrum encompassing a negative band centred at 295 nm and a positive centered at 335 nm. This CD spectrum is not related to BSA alone, which present its intrinsic near-UV-CD signal bellow 300 nm. In short, the CD spectrum of divanillin can be classified as an ICD spectrum caused by its binding BSA. These results are evidence that the achiral divanillin (tropos biphenyl) became chiral (atropos biphenyl) when complexed with BSA. These results are the consequence of the attachment of an optically inactive divanillin inside the chiral microenvironment, which forms the proteins binding sites, leading to induction of chirality. Furthermore, it is an additional and unequivocal confirmation that divanillin was complexed with BSA.

### Application of ICD signal for determination of association constant

Regarding the interaction between proteins and ligands, the existence of ICD signal may contribute significantly for the elucidation of the binding process. Firstly, as described previously, the ICD signal is a direct evidence of the complexation; secondly, the ICD signal can also be used to evaluate the binding affinity. The last feature has advantage upon other spectroscopic techniques. Indeed, as a chiroptical technique, measurements based on ICD is less susceptible to superposition of bands or inner filter effects as usually observed using alterations in fluorescence. For these reasons, we also used the concentration dependent alteration in the ICD signal as an analytical parameter for evaluate the association constant between divanillin and BSA. For that, an analytical rationalization and mathematical derivatization presented by Zsila and collaborates was used [51]. In this methodology, the intensity of the ICD (*θ mdeg*) signal was taken as proportional to the concentration of bound ligand (LP, Eqs 6 and 7). Then, the association constant (*K*_*a*_) was obtained from the non-linear fitting of Eq 8 [51]. Where, *K* is a proportionality constant, *Pt* and *Lt* are the added concentrations of protein and ligand, respectively.

L+P⇋LP (6)

θ(mdeg)=K[LP] (7)

$$\theta \left(mdeg\right)=\frac{K}{2}\left[P_{t}+L_{t}+\frac{1}{K_{a}}-\sqrt{{\left(P_{t}+L_{t}+\frac{1}{K_{a}}\right)}^{2}-4P_{t}L_{t}}\right]$$ (8)

**Fig 4:**
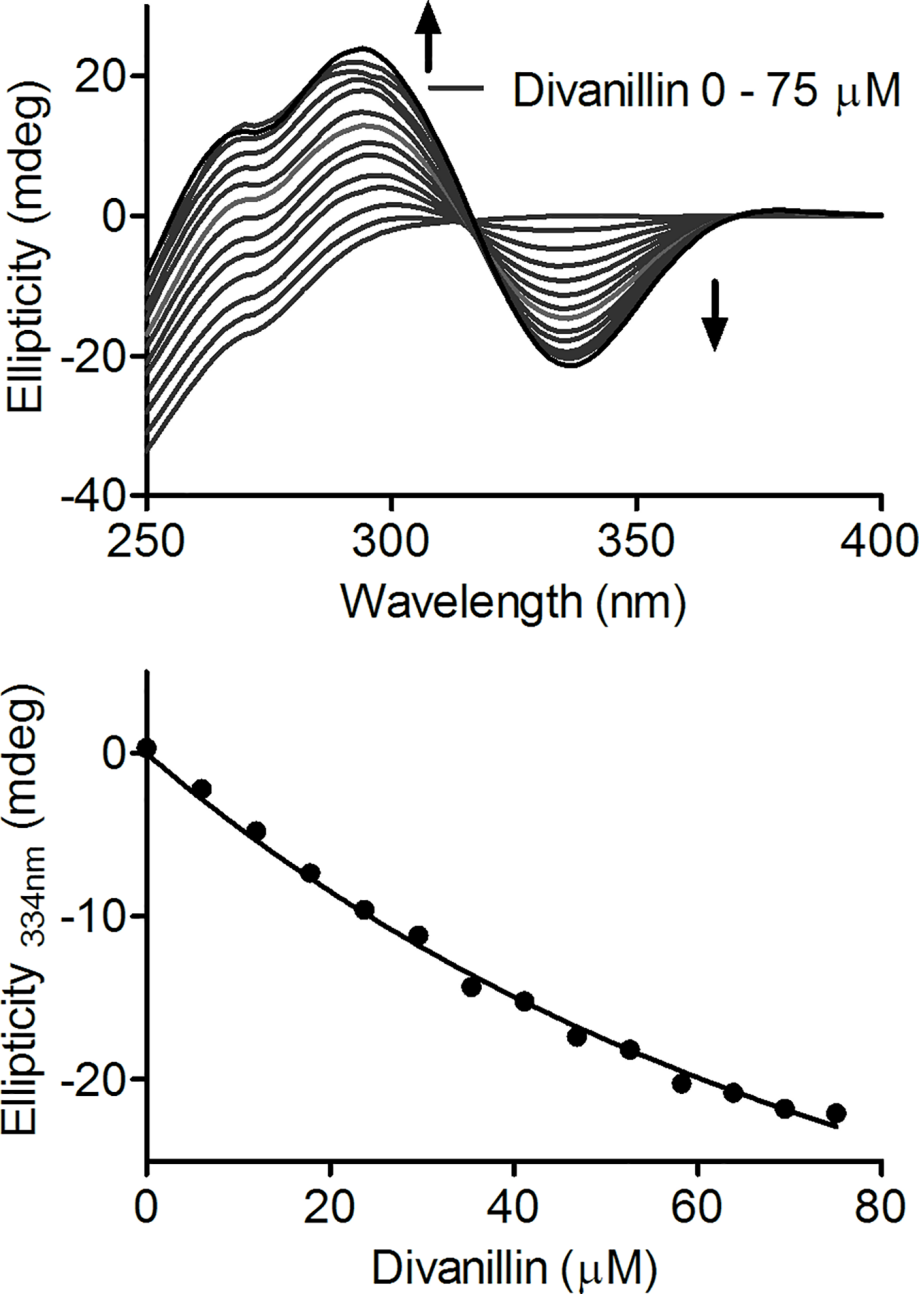
Determination of association constant using ICD on divanillin provoked by its binding in BSA. The mixtures consisted of 30 μM BSA and divanillin (0–75 μM) in 0.05 M phosphate buffer at pH 7.0 at 298 K.

The results depicted in Fig 4 represent the evolution of the ICD signal at increasing amount of divanillin and the non-linear curve fitting (Eq 8), from which *K*_*a*_ (1.3 x 10^4^ mol ^-1^ L) was obtained. As can be observed, this value is about one order of magnitude lower compared to the constant obtained by fluorescence quenching experiment (3.3 x 10^5^ mol^-1^ L). An explanation for this lower value has its fundament in the origin of the ICD signal of divanillin. In fact, it is reasonable to consider that the observed ICD is the result of an enantiomeric excess of a specific conformation of divanillin fixed in the binding sites of BSA. In other words, it is possible that more than one conformation can bind, but one has preference. Hence, the ICD signal of a conformer could be partially concealed by its enantiomeric pair. This fact could explain the lower association constant obtained using this technique. Fig 4 also shows an apparent isodichroic point at 316 nm. This phenomenon is related to the spectral characteristic of divanillin, which has both, positive and negative ICD bands.

### Application of ICD for determination of binding site

Another potential application of the ICD phenomenon is in the elucidation of the binding site of the studied ligand [17]. Thus, we used this chiroptical technique to probe the location of divanillin in BSA. How stated in the introduction section, albumin has two main binding sites (I and II) involved in the transportation of numerous xenobiotics, including anti-inflammatory, antibiotics, antineoplastic agents, etc. For this reason, we chose well-stablished and specific drugs of site I and site II to probe their effects on divanillin ICD signal. The idea was that by adding these specific ligands, a competition could take place leading to the displacement of divanillin from its binding site and causing alterations in the ICD spectrum. The study was performed with two well-established ligands for the site I, warfarin and phenylbutazone, and two ligands for the site II, ibuprofen and naproxen. However, it is worth of note that a pre-condition for the efficacy of the methodology is the absence of intrinsic CD and/or ICD signal provoked by their binding in BSA, or at least that there is not superposition of spectra. To verify these putative interferences, the CD spectra for the complex between BSA and each one of these pharmaceuticals were obtained. From the results in Supporting Information (S5 Fig), it is possible to note that only phenylbutazone was susceptible to induction of chirality in the studied UV-Vis region of interest. For this reason, phenylbutazone was discarded of posterior investigations.

**Fig 5:**
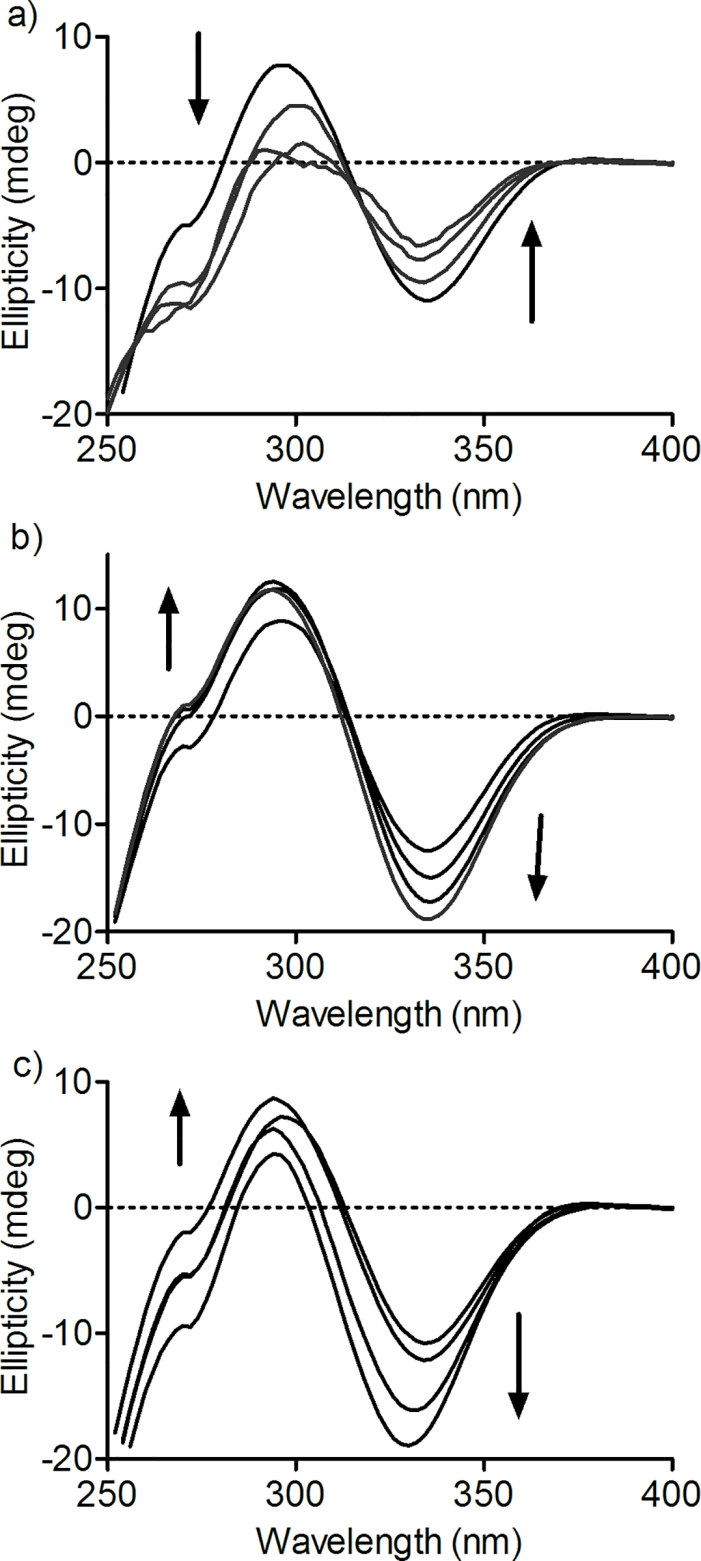
Effect of pharmaceutical drugs on ICD in divanillin. The mixtures consisted of 30 μM BSA, 30 μM divanillin and drugs (0–90 μM) in 0.05 M phosphate buffer at pH 7.0. **(a)** Warfarin (site I), **(b)** ibuprofen (site II) and **(c)** naproxen (site II).

Fig 5A shows the effect on ICD of divanillin provoked by titration with warfarin (site I ligand). How can be observed, the intensity of the ICD spectrum (positive band at 295 nm and negative band at 335 nm) was progressively decreased by the addition of warfarin. Fig 5B shows the effect of ibuprofen (site II ligand), which was exactly the opposite, provoking an increase in the ICD signal. Similar tendency was obtained using naproxen, which is also a site II ligand. In this last case, the effect was still more evident in the negative band. It was also possible to observe a blue-shift in the negative band (Fig 5C). Altogether these results can be interpreted as follows:

1. Considering that divanillin is a site I ligand. Then, the addition of warfarin provoked a partial remotion of divanillin from the protein cavity, indicating a direct competition for this site.
2. The increased divanillin ICD signal provoked by addition of the site II ligands might be an evidence that divanillin does not occupy or occupy with less efficiency this site. In this case, the binding of ibuprofen or naproxen at site II could affect the capacity of divanillin as a ligand of site I, leading to an increased and/or altered signal. Corroborant with that, an increase in the ICD signal of bilirubin (site III) provoked by addition of ethacrynic acid (site II) was interpreted as an allosteric interaction leading increased affinity of the bilirubin at site III [17].

### Confirmation of binding site

**Fig 6:**
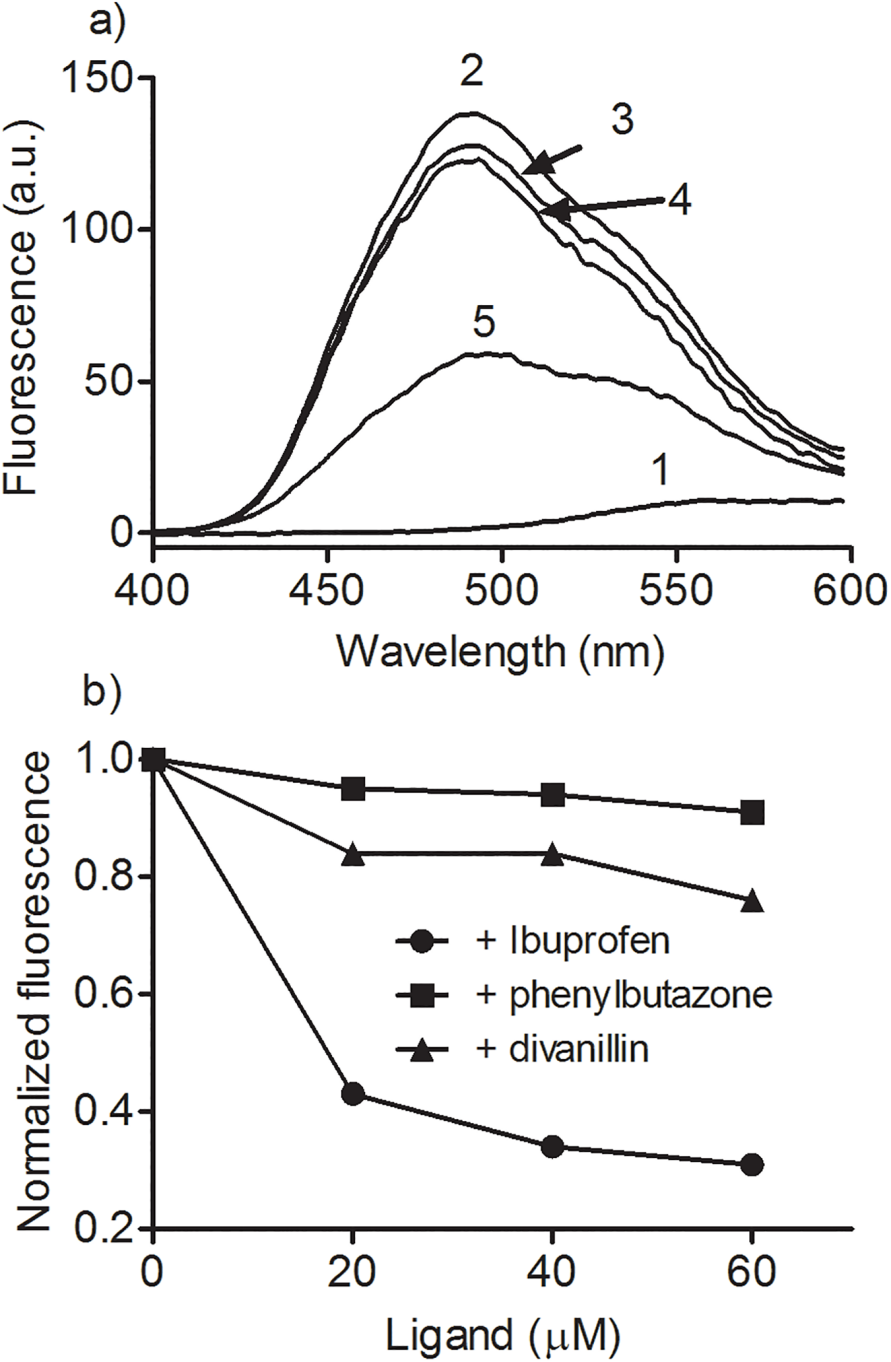
Displacement of dansylproline from BSA by divanillin and pharmaceutical drugs. The mixtures consisted of 10 μM BSA and 20 μM dansylproline in the presence or absence of studied compounds in 0.05 M phosphate buffer at pH 7.0. **(a)** (1) dansylproline, (2) dansylproline + BSA, (3) dansylproline + BSA + phenylbutazone, (4) dansylproline + BSA + divanillin, (5) dansylproline + BSA + ibuprofen. **(b)** Concentration response curves.

The observed effects of the pharmaceutical drugs on ICD signal of divanillin suggested a tendency to a higher affinity of this compound at site I, however, it was not totally conclusive. Hence, two experiments were idealized to further confirmation of the binding site of divanillin in BSA. One of then was the use a fluorescent probe specific for site II. This is the case of dansylproline, a dansylated amino acid that binds in site II and has fluorescence quantum yield increased and shift to a lower wavelength when complexed to albumin [34]. In this experimental approach, the addition of a site II competitor displaces the dansylated amino acid leading to a fluorescence decay. To ascertain of this feature, before the application of divanillin, we studied the effect the addition of phenylbutazone and ibuprofen on the complex BSA:dansylproline. How can be observed in Fig 6, ibuprofen was significantly more effective than phenylbutazone in the displacement of dansylproline from BSA, which proved the efficacy of this compound as a site II marker. Finally, the effect of addition of divanillin was studied and the results reinforced the ICD findings, since the displacement was closer to that observed using phenylbutazone, i.e. divanillin showed preference for site I.

**Fig 7:**
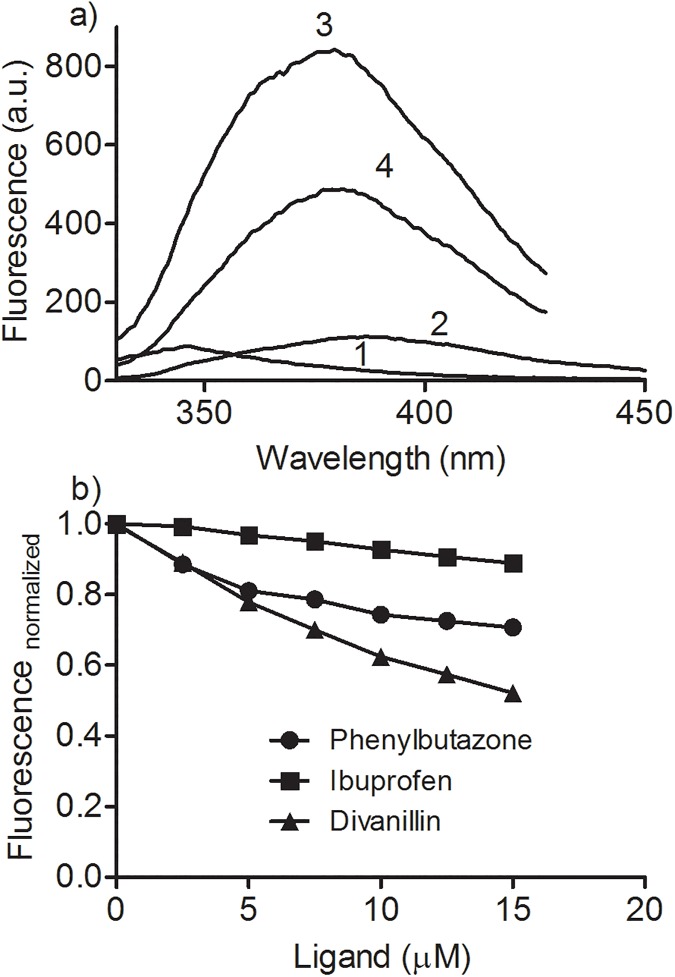
Displacement of warfarin from BSA by divanillin and pharmaceutical drugs. The mixtures consisted of 5 μM BSA and 5 μM warfarin in the presence or absence of studied compounds in 0.05 M phosphate buffer at pH 7.0. **(a)** (1) BSA, (2) warfarin, (3) BSA + warfarin, (4) BSA + warfarin + divanillin. **(b)** Concentration response curves.

Following the same approach, the alteration in the intrinsic fluorescence of warfarin due to its binding in BSA and the subsequent displacement by the addition of divanillin was also studied [33]. The results depicted in Fig 7 show that phenylbutazone was more effective than ibuprofen in the displacement of warfarin, which proved the efficacy of method for characterization of site I. Finally, and corroborant with the previous experiments, divanillin was still more effective that phenylbutazone, reinforcing its preference for site I in BSA.

### Temperature effect on divanillin binding

**Fig 8:**
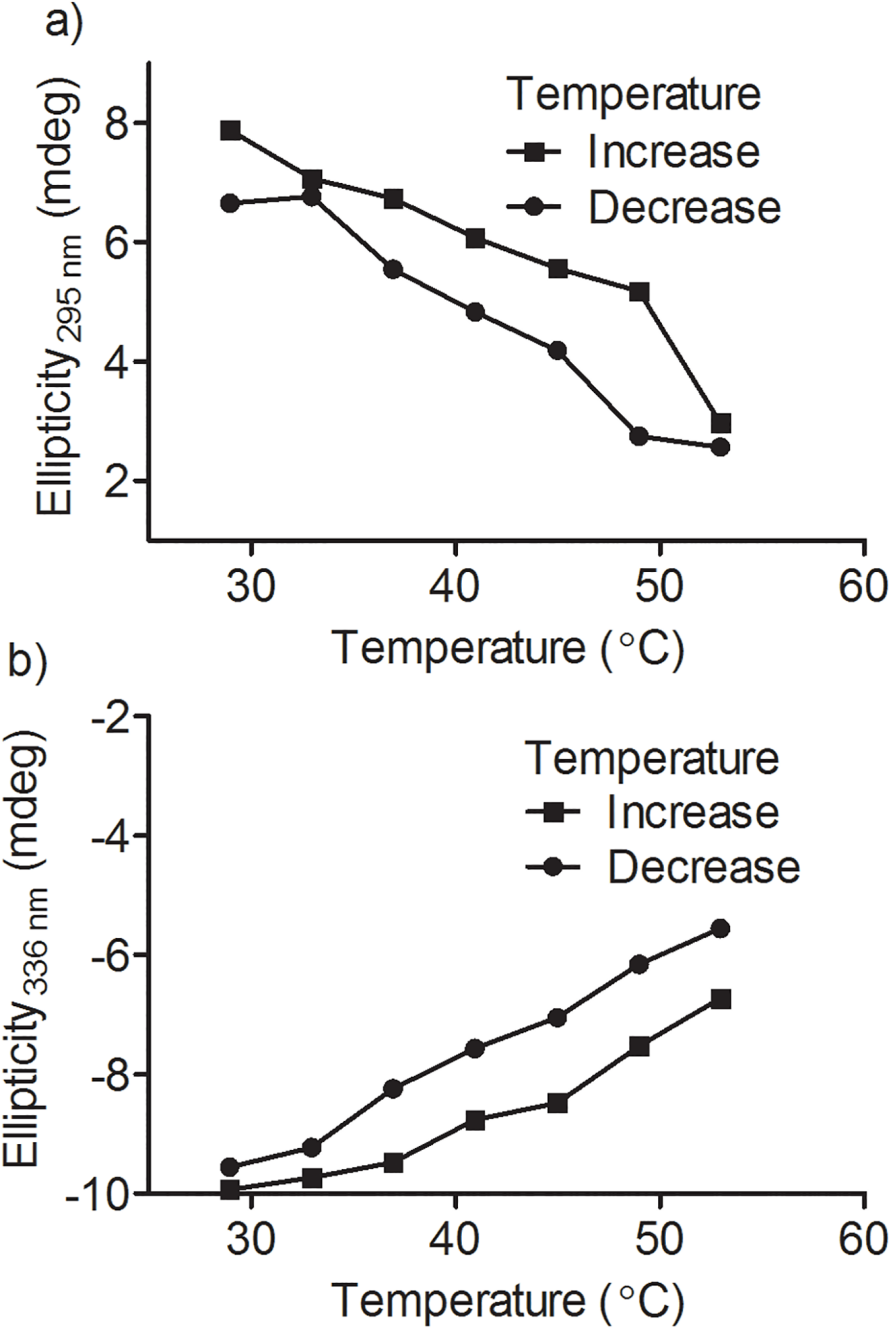
Temperature-dependent reversibility of ICD in divanillin. The mixtures consisted of 30 μM BSA and 30 μM divanillin. The temperature was increased from 25°C to 55°C and returned to 25°C at a rate of 1.0°C/min. **(a)** Ellipticity at 295 nm, **(b)** Ellipticity at 336 nm.

As well stablished, the interaction between protein and ligands is usually weakened, as the temperature is increased [40]. Therefore, if non-covalent forces are responsible by the stabilization of divanillin into the binding site of BSA, the association must be weakened at higher temperature. This phenomenon can be easily studied using ICD, because the origin of the signal is totally dependent of the formation of the complex. In these experiments, the circular dichroism spectrometer was adjusted to increase the temperature from 25°C to 55°C at 1.0°C/min and then return to 25°C at the same rate. The ICD was monitored at both positive and negative bands. Fig 8 shows that the ICD signal decreased over this temperature range and returned to almost the same initial value when the temperature returned back to 25°C. In short, these results reinforce the existence of a complex between BSA and divanillin and the reversibility of the binding.

### Docking studies

**Fig 9:**
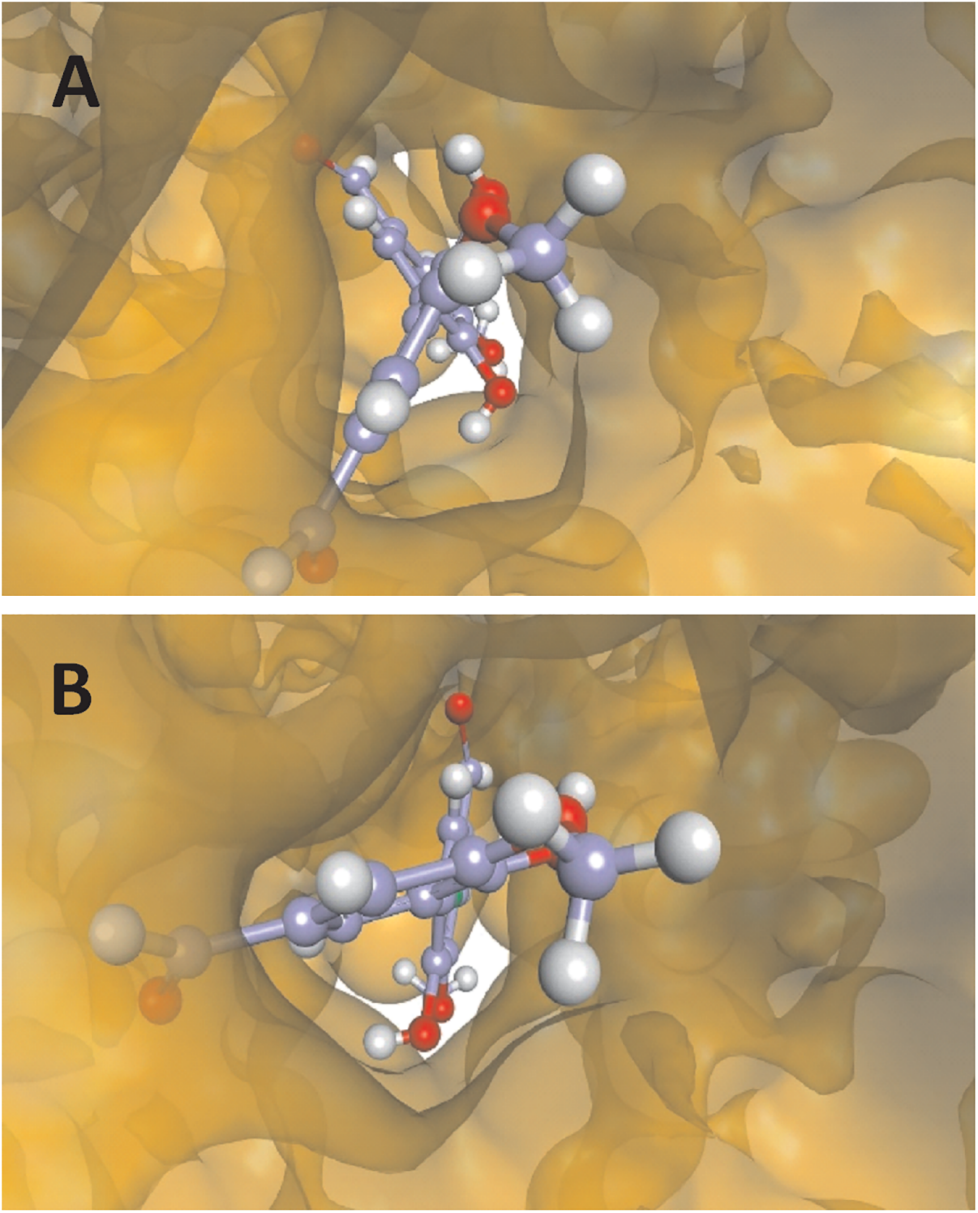
The BSA site I cavity complexed with divanillin. This cavity is like a cylinder open on both sites (A and B).

**Fig 10:**
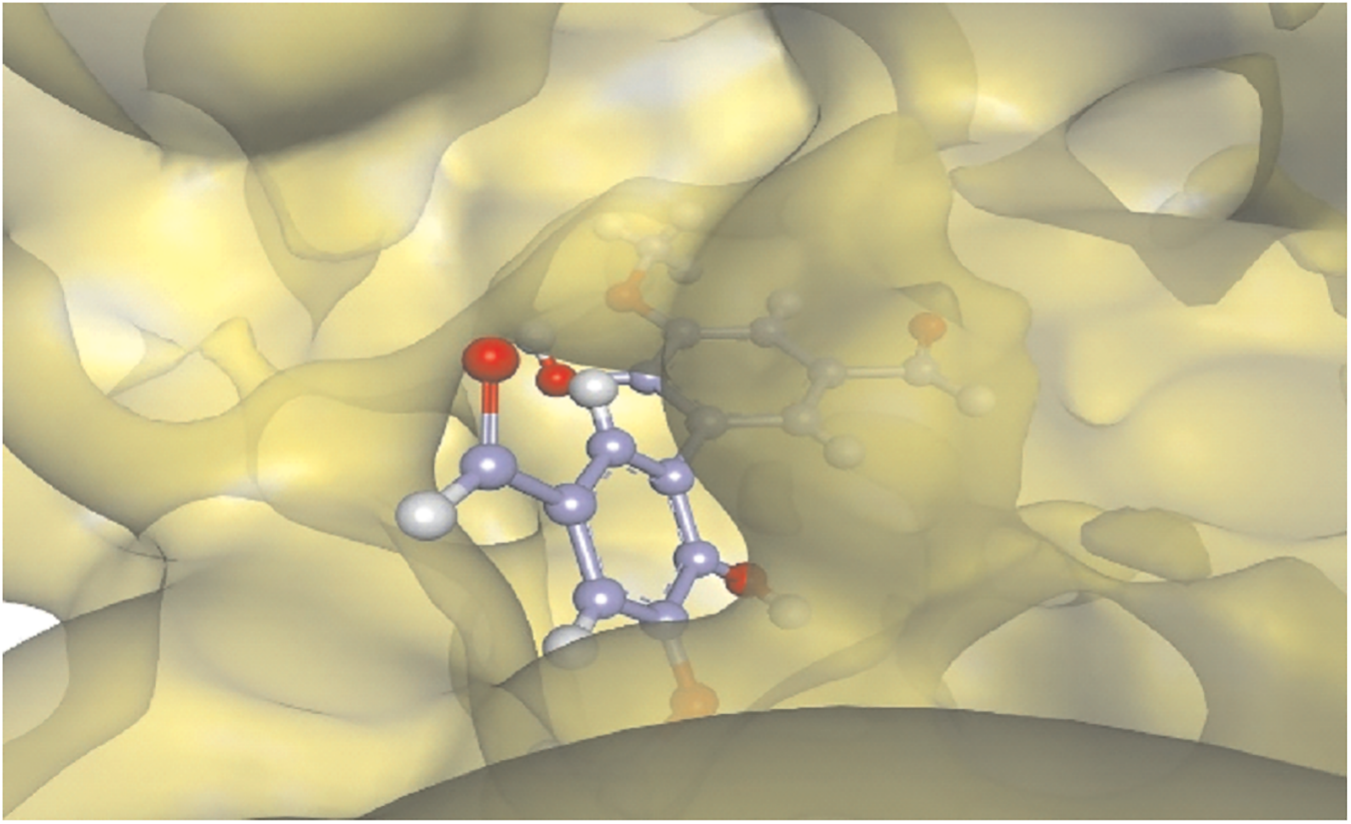
The BSA site II cavity complexed with divanillin. This cavity is open in one side and closed in other.

The existence of an ICD spectrum for divanillin implies that it must have a non-zero dihedral angle around the single bond that links the phenyl rings. Therefore, our next step was to perform docking studies to obtain the conformations of divanillin bound to site 1 and 2 of BSA. For these studies, the crystallographic structure used was that of BSA in complex with naproxen available in PDB (access code 4OR0). In this crystallographic structure, naproxen occupies three different sites in BSA, named naproxen site 1 (NPS1), naproxen site 2 (NPS2) and naproxen site 3 (NPS3). As stablished by the authors, NPS3 corresponds to Sudlow's site I and NPS1 to Sudlow's site II, which are the binding sites of interest here. NPS 2 is an alternative site for naproxen and was not considered in our study [37]. From this crystallographic structure, we found that the closest amino acids to divanillin at site I were Arg194, Trp213 and Arg198 and for site II the closest amino acids were Arg409, Tyr 410 and Lys 413. The scores for the binding and the main intermolecular forces involved in the stabilization of divanillin in both sites are shown in Table 3. How can be seen from the stabilization energies, the docking simulations showed that divanillin has preference for site I, -63.1 kJ mol^-1^, compared to site II, -59.7 kJ mol^-1^. Despite the difference in the absolute values, these score values are consistent with the experimentally determined binding energy ΔG°, -31.5 kJ mol^-1^) and reinforce the thermodynamic favorability of the complexation. The higher score for site I is also in agreement with our experimental results, which showed the preference of divanillin for this site. Fig 9 shows the docking pose for divanillin complexed with BSA in the cavity that constitute the site I. This site is like a cylinder open on both sides where the divanillin is aligned with the main axis of the cylinder with the two rings almost perpendicular one to the other. Site II, which is a cavity open in one side end closed in the other, complexed with divanillin is showed in Fig 10.

**Table 3:** Score and molecular interactions between divanillin and the amino acid residues in BSA binding sites obtained by docking simulations.

Binding Site	Molecular Interactions with Amino Acids							Score (kJ mol^-1^)	
**I**	R194	R198	W213	S343	D450				-63.1
	vdw	HB	NH^…^π	HB	HB				
**II**	N390	R409	Y410	L429	G433	L452	S488		-59.7
	HB	CH^…^π	HB	vdw	vdw	vdw	vdw		

Van der Waals (vdw), hydrogen bond (HB), π interaction with amino group (NH ^…^ π), π interaction with CH group (CH ^…^ π).

### Theoretical studies: Dihedral conformation of divanillin

**Fig 11:**
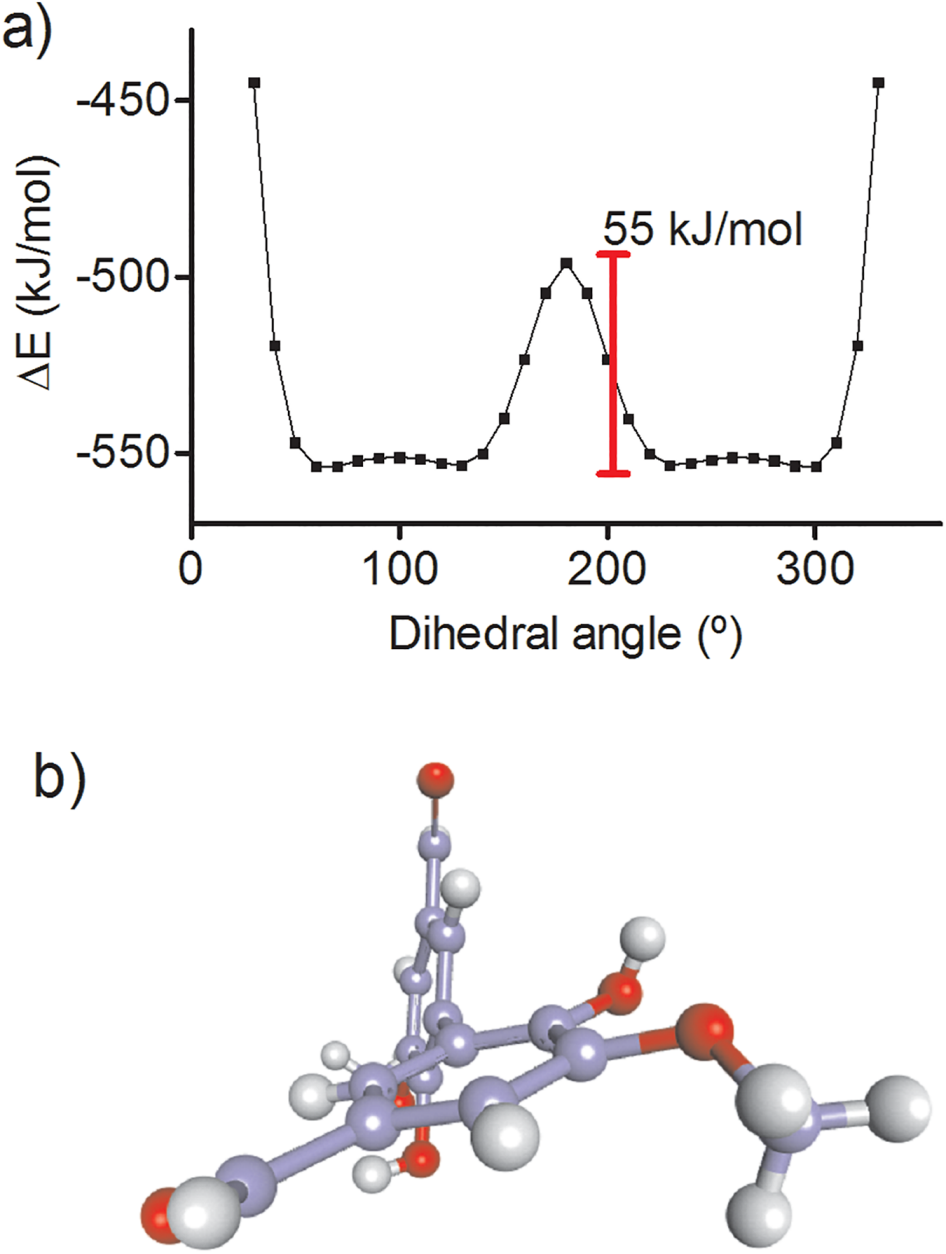
a) Energy of the conformations of divanillin around of the single bound that links the aromatic rings obtained by M06-2X method along with the 6-31G(d) basis set. b) Minimum energy conformation of divanillin in site I obtained by docking simulation (dihedral angle 242°).

An ICD signal provoked by the binding of divanillin in BSA is related to the induction of axial chirality in this di-ortho substituted biphenyl. It means that divanillin, a tropos biphenyl, behaves as an atropos when attached into the site I and, more importantly, some degree of stereoselectivity must be present, otherwise no ICD signal should appear, since a conformation could be concealed by its enantiomer. Therefore, there must be a preferential stabilized conformation with a non-zero dihedral angle around the single bond that links the two phenyl rings. Aiming to elucidate this minimum energy conformation, theoretical studies were performed searching for the most stable conformations of divanillin and the results were compared with those obtained by docking simulations. Density Function Theory (DFT) using the M06-2X functional along with the 6-31G(d) basis set was initially used to search for the most stable conformations adopted by divanillin in the gas phase. The energy for each conformation of divanillin was obtained and plotted as a function of the dihedral angle (δ dihedral angle, the angle between the two benzene rings). The potential energy surface (PES) which consists of single point energy evaluations over a rectangular grid involving the Cartesian coordinates of divanillin atoms was obtained. The interval and step size were 0 to 360 and 10 degrees, respectively. In this methodology of calculation, the solvent, water, was included through the Polarised Continuum Medium (PCM) model, and the harmonic vibrational frequency for characterisation of the stationary points was obtained [52]. Fig 11 shows the energy scan as a function of the dihedral angle. How can be seen the energy barrier that impedes the interconversion of the atropisomers was about 55 kcal/mol. This value is significantly less than 98 kcal/mol, which has been attributed as a minimum energy barrier for the existence of atropisomerism [53]. In other words, this theoretical result is consistent with our experimental finding that divanillin in aqueous solution behaves as a tropos system. However, inside the protein this barrier must be higher, impeding the free conversion of the enantiomers, i.e., in the cavities that constitute the binding site of BSA, divanillin became an atropos system.

The four minima energy conformations for divanillin were verified at dihedral angles of about 60°, 120°, 230° and 300° (Fig 11A). Interestingly, the conformation adopted for divanillin at site I obtained in the docking simulation was 242°, which is quite close to 230°, one of the minimum found in the DFT studies (Fig 11B). These results suggest that the intermolecular interactions with the amino acids residues at the binding site (Table 3) are essential for stabilization of this specific conformation, otherwise the other conformers, which has similar energy, could also bind and consequently to vanish the ICD signal.

### Simulation of circular dichroism spectrum of divanillin

**Fig 12:**
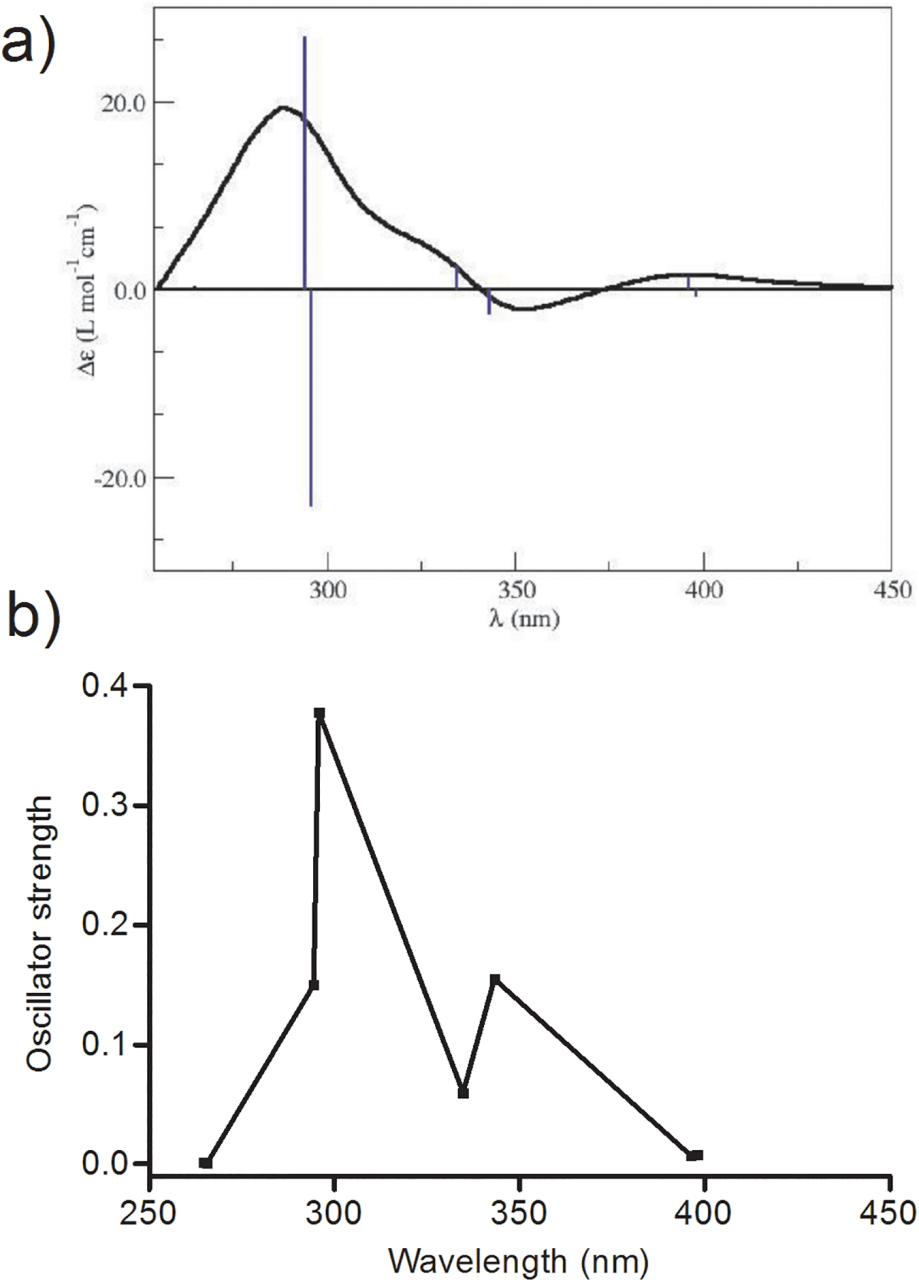
(a) Simulated ICD spectrum of the lower energy conformation of divanillin complexed with HSA (dihedral angle 242°). (b) Oscillator strength as a function of the wavelength for the seven first excited states of divanillin.

Once found the most stable conformation of divanillin in site I of BSA, the next step was the simulation its ICD spectra. The computer simulation was performed at the TDDFT level of theory using CAM-B3LYP, the hybrid exchange-correlation functional Coulomb-attenuating method, and the 6–311++G(2d,p) basis set [54]. The solvent, ethanol, was used implicitly, through the use of the dielectric constant (ε = 24.852). Fig 12 shows the simulated ICD spectrum of the lower energy divanillin conformer obtained in the docking study (dihedral angle 242°). The output of the computer simulations of ICD was obtained as a list of rotational strengths Ri at each discrete transition frequencies, and then converted in an ICD spectrum, i.e., at each rotational strength was associated with a band-shape function whose intensity is proportional to the value of Ri and then proceed to sum over all bands obtained [55]. Upon analysis of Fig 12A, we can inferred that the major contribution to the ICD spectrum of divanillin signal correspond to three bands 295.87, 334.74, and 396.26 nm. These results are in good agreement with the experimental ones. An exception was the band at 396.26 nm, which was not observed experimentally; in this case, we can infer that the characteristic of the computer model is not capable of a complete description, with details, of the environmental of the ligand site inside the protein. Further calculations can been done to improve the theoretical model in the description of the complex interaction scenario composed of the divanillin molecule and the protein site as, for example, a Quantum Mechanics approach in conjunction with a Molecular Mechanics description of the system of interest.

In the Fig 12B we present the electronic transitions between the ground state and seven excited states in the wavelength range of 264.80 to 398.06 nm and the respective oscillator strength for these transitions. We can verify that the oscillator strength increases as we approach the region of 300 nm, being equal to 0.3779 for the wavelength equal to 295.87 nm; this region is where we can observe the highest absorption of divanillin in the ECD spectrum. This value of the oscillator force is associated to the transition between the ground state and the sixth state excited with singlet character. As the wavelength increases the oscillator strength decreases. This electronic transition occurs between the ground state and the third singlet state at the wavelength of 343.27 nm with oscillator strength equal to 0.1548. This transition is associated with the absorption band in the region of 350 nm in the ECD spectrum of divanillin. The absorption band in the region of 400 nm in the ECD spectrum is related to the transitions between the ground state and the second and third singlet excited state with an oscillator force equal to 0.0074 and 0.1548, respectively.

## Conclusions

By interacting with the chiral cavities of the BSA, divanillin became a atropos biphenyl, i.e., the free rotation around the single bound that links the aromatic rings was impeded. This phenomenon can be explained considering the interactions with amino acid residues in the binding site of the protein. The chirality of divanillin complexed to BSA was evidenced by the generation of an ICD spectrum. The efficiency of complexation was proven by the high association constant between the divanillin and BSA. The ICD signal in divanillin was useful for determination of site I as the main binding site of this ligand in BSA. The ICD signal was also useful for confirmation of the high association constant and to demonstrate the reversibility of the binding of divanillin with BSA. The conformation of divanillin obtained for docking simulation (dihedral angle 242°) was used to calculate the ECD spectrum using TDDFT. The theoretical spectrum showed good similarity with the experimental. Considering the potential pharmacological application of divanillin, these findings will be helpful for researchers interested in the pharmacological properties of this compound.

## Supporting information

S1 Fig HPLC analysis of the synthesized diapocynin and comparison with its precursor vanillin. Vanillin and divanillin 100 μM in 0.05 M phosphate buffer 0.05 M pH 7.0.
(DOCX)

S2 Fig NMR spectra of divanillin obtained using DMSO- D6 as solvent and internal reference for ^1^H and ^13^C (Bruker DRX 400 spectrometer, MA, USA). (DOCX)

S3 Fig Determination of the association constant between BSA and vanillin. Double logarithmic fitting for determination of the association constant. The results are the average and SD of experiments performed in triplicate. Experimental condition: 5 μmol L^-1^ BSA in the absence or presence of vanillin (0–30 μmol L^-1^) in 0.05 mol L^-1^ phosphate buffer pH 7.0 at 298 K (λ_ex_ = 295 nm, (λ_em_ = 343 nm).
(DOCX)

S4 Fig Determination of thermodynamic parameters for divanillin binding to BSA. Van`t Hoff plot.
(DOCX)

S5 Fig Investigation of the existence of ICD provoked by the binding of BSA with the pharmaceutical drugs used for characterization of binding sites. Experimental condition: 30 μmol L^-1^ BSA in the absence or presence of pharmaceutical drug (30 μmol l L^-1^) in 0.05 mol L^-1^ phosphate buffer pH 7.0 at 298 K.
(DOCX)
