# Supplementary material for: Induction of axial chirality in divanillin by interaction with bovine serum albumin
Source: PLoS One. 2017 Jun 2;12(6):e0178597. doi: 10.1371/journal.pone.0178597 (PMC5456067; doi:10.1371/journal.pone.0178597)
Supplement: S1 Fig — Vanillin and divanillin 100 μM in 0.05 M phosphate buffer 0.05 M pH 7.0. (DOCX) [file pone.0178597.s001.docx]

**S1 Fig:** HPLC Analysis of the synthesized diapocynin and comparison with its precursor vanillin. Vanillin and divanillin 100 µM in 0.05 M phosphate buffer 0,05 M pH 7.0.
