# Supplementary material for: Induction of axial chirality in divanillin by interaction with bovine serum albumin
Source: PLoS One. 2017 Jun 2;12(6):e0178597. doi: 10.1371/journal.pone.0178597 (PMC5456067; doi:10.1371/journal.pone.0178597)
Supplement: S2 Fig — (DOCX) [file pone.0178597.s002.docx]

**S2 Fig**: NMR spectra of divanillin obtained using DMSO- D_6_ as solvent and internal reference for ^1^H and ^13^C (Bruker DRX 400 spectrometer, MA, USA).
