# Supplementary material for: Induction of axial chirality in divanillin by interaction with bovine serum albumin
Source: PLoS One. 2017 Jun 2;12(6):e0178597. doi: 10.1371/journal.pone.0178597 (PMC5456067; doi:10.1371/journal.pone.0178597)
Supplement: S3 Fig — Double logarithmic fitting for determination of the association constant. The results are the average and SD of experiments performed in triplicate. Experimental condition: 5 μmol L-1 BSA in the absence or presence of vanillin (0–30 μmol L-1) in 0.05 mol L-1 phosphate buffer pH 7.0 at 298 K (λex = 295 nm, (λem = 343 nm). (DOCX) [file pone.0178597.s003.docx]

**S3 Fig**. Determination of the association constant between BSA and vanillin. Double logarithmic fitting for determination of the association constant. The results are the average and SD of experiments performed in triplicate. Experimental condition: 5 μmol L^-1^ BSA in the absence or presence of vanillin (0 - 30 μmol L^-1^) in 0.05 mol L^-1^ phosphate buffer pH 7.0 at 298 K (λ_ex_ = 295 nm, λ_em_ = 343 nm).
