# Supplementary material for: Induction of axial chirality in divanillin by interaction with bovine serum albumin
Source: PLoS One. 2017 Jun 2;12(6):e0178597. doi: 10.1371/journal.pone.0178597 (PMC5456067; doi:10.1371/journal.pone.0178597)
Supplement: S4 Fig — Van`t Hoff plot. (DOCX) [file pone.0178597.s004.docx]

**S4 Fig**. Determination of thermodynamic parameters for divanillin binding to BSA. Van`t Hoff plot.
