# Supplementary material for: Induction of axial chirality in divanillin by interaction with bovine serum albumin
Source: PLoS One. 2017 Jun 2;12(6):e0178597. doi: 10.1371/journal.pone.0178597 (PMC5456067; doi:10.1371/journal.pone.0178597)
Supplement: S5 Fig — Experimental condition: 30 μmol L-1 BSA in the absence or presence of pharmaceutical drug (30 μmol l L-1) in 0.05 mol L-1 phosphate buffer pH 7.0 at 298 K. (DOCX) [file pone.0178597.s005.docx]

**S5 Fig.** Investigation of the existence of ICD provoked by the binding of BSA with the pharmaceutical drugs used for characterization of binding sites. Experimental condition: 30 μmol L^-1^ BSA in the absence or presence of pharmaceutical drug (30 μmol L^-1^) in 0.05 mol L^-1^ phosphate buffer pH 7.0 at 298 K.
